# Undergraduate oncology education in Sudanese public medical schools; a national cross-sectional study

**DOI:** 10.1186/s12909-023-04883-0

**Published:** 2023-12-10

**Authors:** Salma S. Alrawa, Ammar Elgadi, Esraa S. A. Alfadul, Shahd Alshikh, Nazik Hammad, Abdelhafeez Abdelhafeez

**Affiliations:** 1 Faculty of Medicine, University of Khartoum, Khartoum, Sudan; 2 Faculty of Medicine, Alzaiem Alazhari University, Khartoum, Sudan; 3 Department of Oncology, Queen’s University, Kingston, ON Canada; 4 Faculty of Medicine, University of Gezira, Gezira, Sudan

**Keywords:** Medical education, Curriculum, Cancer, Oncology, Sudan, Developing countries

## Abstract

**Background:**

Cancer was the fifth leading cause of death in Sudan general hospitals in 2020, and its incidence is increasing. Medical students’ cancer education is key in cancer control. Evaluating the current education is the first step in optimizing it. The aim of this study was to assess undergraduate oncology education in Sudan public medical schools as reflected by the graduates of the year 2021–2022.

**Method:**

This was a cross-sectional institution-based study. A validated online questionnaire was sent between 8 September and 11 November 2022 to graduates who were selected using a stratified random sampling technique from 17 Sudan public medical schools. The data were collected using Google Forms and analyzed using R software version 4.2.2 and Microsoft Excel 2022.

**Results:**

A total of 707 graduates completed the questionnaire. They reported generally poor exposure to oncology. Palliative and radiation oncology in addition to chemotherapy daycare units were never attended by 76.0%, 72.0%, and 72.0% of graduates, respectively. The massed oncology curriculum was associated with increased hours of lectures dictated to medical (*p* = 0.005), radiation (*p* < 0.001), and palliative oncology (0.035). It was associated with an increased likelihood of assessment in breaking bad news (*p* < 0.001), counseling cancer patients (*p* = 0.015), and oncology-related knowledge (*p* < 0.001). The massed curriculum was associated with a decrease in interest in pursuing an oncology career (*p* = 0.037). Students were generally confident in their oncology competencies, and no difference was observed in relation to the curriculum approach (*p* > 0.05).

**Conclusion:**

This study reflected poor exposure to oncology at the undergraduate level in Sudanese public medical schools. The massed oncology curriculum was associated with formal assessment of oncology-related competencies and better exposure to some disciplines, such as radiation and palliative oncology. Nonetheless, it was associated with decreased interest in an oncology career. In spite of the poor exposure, graduates were confident in their skills in oncology-related competencies. Further objective analysis of competence is needed.

**Supplementary Information:**

The online version contains supplementary material available at 10.1186/s12909-023-04883-0.

## Background

Cancer is a leading cause of death and was responsible for approximately 10 million deaths in 2020 [1]. It causes 20% of overall global mortality, and 65% of these deaths occur in lower- and middle-income countries (LMICs) [2]. According to a 2020 Sudan statistical report, cancer is the fifth leading cause of death in Sudan [3]. Global Cancer Incidence, Mortality and Prevalence (GLOBOCAN) reported that the 5-year prevalence of cancer in Sudan is 48 694 cases and that the risk of dying from cancer is 6.4% [1]. The burden of cancer and non-communicable diseases is increasing, and that of communicable diseases is not improving, which maximizes the challenge [4].

Cancer diagnosis, emergency management, and referral are the responsibilities of general practitioners [5]. Inadequate training at medical school negatively impacts multidisciplinary care [6], which may harm patients. Medical schools prepare their graduate students with the basic requirements for practice [7], which necessitates a change in curriculum with changes in science, disease burden, and patients’ needs [5].

In Sudan, healthcare services are provided in three levels namely; primary, secondary, and tertiary. Primary care providers serve as the point of entry to the system and are responsible for the control of non-communicable diseases [8]. Some of the recognized weaknesses of the Sudanese health care system include scarcity of continuing medical education programs and postgraduate training [8]. This highlights the effect of undergraduate training on general practitioners’ practice. The WHO has highlighted the importance of training general practitioners to ensure early cancer diagnosis [9].

Recognizing the role of general practitioners in cancer care has led to the development of an oncology curriculum in several high-income countries (HICs), including Canada [10], European countries [11], and Australia [12]. As part of the course provided by the European School of Oncology (ESO), prof. N. Pavlidis revised the developing and international experience with undergraduate oncology and emphasized the need for oncology education [13]. Low- and middle-income countries (LMICs) were largely absent from the picture. A review conducted by Amgad et al. in developing countries revealed the lack of cancer-related knowledge among graduates and practicing physicians [14].

The higher committee for cancer control in Sudan has recommended curriculum amendments to ensure cancer education at both undergraduate and postgraduate level [15]. This recommendation was not followed with action. Among more than 72 medical schools in Sudan, only four medical schools have a formal undergraduate oncology course. The rest of medical schools teach oncology as part of other subjects and the hours dictated to oncology as well as oncology related objectives are not clearly described. Unfortunately, we don’t have agreed upon competencies for graduates in Sudan [16]. The aims of this study were to describe the undergraduate oncology education in Sudan as reflected by 2021–2022 graduates, to compare the exposure to oncology between schools with massed oncology education – as reflected by having a separate oncology course - and those with spaced out oncology education – where oncology is taught as part of other courses - and to report graduates’ perceived competence in oncology and the factors associated with it.

## Methods

### Study design and setting

This was a cross-sectional institution-based study. We included the public medical schools in Sudan which had more than 100 graduates in the year 2021–2022. Sudan has more than 72 medical schools distributed across the country and almost two thirds of them are located in the central region [17] A total of 30 public medical schools were identified from the “admission guide for Sudanese universities 2021” available online from the Sudanese ministry of higher education website. Nine schools were recently established and had no graduates up to the date of the start of the study. From the remaining 21 universities, 4 had less than 100 graduates in the year 2021–2022. The remaining 17 universities were included in the study and they are: University of Albutana, Bakht Alruda University, Sennar University, Shendi University, University of Gadarif, Alfashir University, University of Elimam El mahdi, University of Kassala, Red Sea University, University of Kordofan, Alzaiem Alazhari University, University of Bahri, Omdurman Islamic University of males, Omdurman Islamic University of females, Gezira University and University of Khartoum.

### Study participants

We included all 2021–2022 graduates from the chosen universities who agreed to participate in this study. Non-Sudanese graduates as well as repeaters were excluded. The sample size was calculated by epi-info app version 7 using the known population formula, taking into consideration the stratified sampling technique. The target population was 2912 graduates. Using a design effect of 2, the sample size was 678. Taking a response rate of 80%, the final sample size was 847. These were randomly selected from the lists of 2021–2022 graduates of the selected medical schools. Participation was totally voluntary and informed consent was taken at the start of the questionnaire. Among the targeted sample a total of 707 graduates filled the questionnaire. Supplementary Table 1.

### Data collection method

The data were collected using an online self-administered questionnaire that was distributed by the principal investigator and collaborators to the selected graduates using What’s up and Telegram platforms. The data were collected between 8 September and 11 November 2022. The questionnaire was developed by the principal investigator with a main reliance on the questionnaire developed by Bravery et al. [18], taking into consideration the competencies highlighted in the ideal oncology curriculum [12], Canadian oncology curriculum [10] andcurriculum of oncology in Europe [11]. The questionnaire was validated by a group of medical, surgical and radiation oncologists as well as specialists in medical education. It was piloted in a sample of 40 medical students from targeted universities for clarity, practicality and reliability. Cronbach’s alpha was found to be 0.939, which indicates excellent reliability. The questionnaire had four parts:

The first part explored students’ attitude toward oncology and its education. It had questions on interest in pursuing a career in oncology, satisfaction with undergraduate oncology education, preferred educational approach and agreement with having a national oncology curriculum.

The second part was about self-reported confidence in various oncologic competencies identified from international and local curricular. The panel agreed on 12 relevant competencies namely; advising patients on prevention and screening, recognizing alarming signs and symptoms, requesting appropriate investigations and interpreting their results, knowledge of urgent referral pathways, managing emergencies, taking care of dying cancer patients and of cancer survivors. Other competencies include breaking bad news, discussing basic etiology and pathophysiology of cancer and interpreting oncology related research papers.

The third part assessed exposure to essential experience in oncology namely; observing multidisciplinary care, observing shared decision making and assessing common cancers in Sudan. Common cancers were identified from a study on prevalence of cancer in Sudan [19].

The fourth part was related to graduates’ assessment in oncology related skills and knowledge like history taking, examining cancer patients, breaking bad news and counseling.

### Data management and analysis

Questionnaires and records were refined and managed carefully. Data were cross-checked for duplication, inaccurate entries, and completeness. The collected data were exported to a Microsoft Excel database. They were cleaned and checked for completion. Analysis was performed using R software version 4.2.2 and Microsoft Excel 2022. Descriptive statistics were used. The normality of the data was tested using histograms and the Kolmogorov‒Smirnov test. Frequency and percentages were used for categorical variables.

A two-sample t test and analysis of variance (ANOVA) were used to identify the predictors of self-reported oncological competence. The chi-square test was used to determine the association between educational approach and undergraduate oncology exposure as well as attitude toward oncology. Stacked bar and heatmap were used to visualize the data.

## Results

### Attitude toward oncology and its education

In this study, 707 participants took part, with approximately one-third expressing their intent to pursue a career in oncology. Almost 90% of participants supported the inclusion of oncology in Sudanese university curricula, including the establishment of a national oncology undergraduate curriculum, and 46% favored spaced-out oncology education (Table 1).

**Table 1:** Attitude toward oncology among undergraduate medical students

Characteristic	N = 707^1^
**Are you interested in pursuing an oncology career?**	
Yes	229 (32%)
No	272 (38%)
Not sure	206 (29%)
**Cancer should not be on the list of Ministry of Health priorities**	
Strongly agree	26 (3.7%)
Agree	43 (6.1%)
Neutral	32 (4.5%)
Disagree	236 (33%)
Strongly disagree	370 (52%)
**Oncology should be an integral part of undergraduate curriculum in Sudanese Universities**	
Strongly agree	309 (44%)
Agree	318 (45%)
Neutral	60 (8.5%)
Disagree	13 (1.8%)
Strongly disagree	7 (1.0%)
**We should develop a national oncology undergraduate curriculum**	
Strongly agree	268 (38%)
Agree	363 (51%)
Neutral	61 (8.6%)
Disagree	13 (1.8%)
Strongly disagree	2 (0.3%)
**In your opinion which curriculum approach of undergraduate oncology is better?**	
Massed education	306 (43%)
Spaced out education	326 (46%)
No difference	75 (11%)
**Are you satisfied with the quality of oncology education at your medical school?**	
Very dissatisfied	49 (7.0%)
Dissatisfied	184 (26%)
Neutral	248 (35%)
Satisfied	177 (25%)
Very satisfied	45 (6.4%)
Missing	4

^1^n (%)

### Undergraduate oncology exposure between universities with a massed oncology curriculum and those with a spaced-out curriculum

Regarding the professional aspect, 398 (56.3%) of the participants did not observe shared decision making between patients and doctors, and 379 (53.6%) did not observe multidisciplinary care. There was no statistically significant difference between universities with a massed curriculum and those with a spaced out curriculum in this regard. (*p* > 0.05). When asked about assessment, students were less likely to be assessed in breaking bad news (365, 51.7%) or counseling cancer patients (377, 53.4%). It is worth noting that graduates of universities with massed oncology curricula were more likely to be assessed in terms of breaking bad news (*p* < 0.001), counseling cancer patients (0.015), and oncology-related knowledge (*p* < 0.001) (Table 2).

**Table 2:** Association between the type of oncology curriculum and oncology exposure, interest, and satisfaction

		Oncology curriculum		
Characteristic	Overall, N = 707^1^	Massed N = 172^1^	Spaced-out, N = 535^1^	*p*-value^2^
**Interest in pursuing an oncology career**				**0.037**
Yes	229 (32%)	42 (24%)	187 (35%)	
No	272 (38%)	74 (43%)	198 (37%)	
Not sure	206 (29%)	56 (33%)	150 (28%)	
**Satisfaction with the quality of oncology education**				**< 0.001**
Very dissatisfied	49 (7.0%)	6 (3.5%)	43 (8.1%)	
Dissatisfied	184 (26%)	30 (17%)	154 (29%)	
Neutral	248 (35%)	61 (35%)	187 (35%)	
Satisfied	177 (25%)	54 (31%)	123 (23%)	
Very satisfied	45 (6.4%)	21 (12%)	24 (4.5%)	
Missing	4	0	4	
**Observing multidisciplinary care**				0.8
No	379 (54%)	91 (53%)	288 (54%)	
Yes	328 (46%)	81 (47%)	247 (46%)	
**Observing the shared decision-making process between doctors and cancer patients**				> 0.9
No	398 (56%)	97 (56%)	301 (56%)	
Yes	309 (44%)	75 (44%)	234 (44%)	
**Assessment of history taking from cancer patient**				0.5
No	115 (16%)	25 (15%)	90 (17%)	
Yes	592 (84%)	147 (85%)	445 (83%)	
**Assessment of examining a cancer patient**				0.5
No	190 (27%)	50 (29%)	140 (26%)	
Yes	517 (73%)	122 (71%)	395 (74%)	
**Assessment of breaking bad news to cancer patients**				**< 0.001**
No	364 (51%)	68 (40%)	296 (55%)	
Yes	343 (49%)	104 (60%)	239 (45%)	
**Assessment of counseling cancer patients**				**0.015**
No	328 (46%)	66 (38%)	262 (49%)	
Yes	379 (54%)	106 (62%)	273 (51%)	
**Assessment of oncology knowledge**				**< 0.001**
No	196 (28%)	26 (15%)	170 (32%)	
Yes	511 (72%)	146 (85%)	365 (68%)	
**Total number of assessed cancer cases**				0.14
10 times or less	117 (17%)	30 (17%)	87 (16%)	
11–20 times	422 (60%)	93 (54%)	329 (61%)	
21–30 times	125 (18%)	33 (19%)	92 (17%)	
31 times or more	43 (6.1%)	16 (9.3%)	27 (5.0%)	

^1^n (%)

^2^Pearson’s Chi-squared test

Regarding hours devoted to oncology, palliative and radiation oncologyin addition to chemotherapy daycare units received the least attention, with 474 (76.0%). A total of 509 (72.0%) and 509 (72.0%) students reported zero hours attending clinical rounds or skill labs in these disciplines, respectively. Pediatriconcology also lagged behind, and 360 (51.0%) of the participants reported zero hours attending pediatric oncology rounds or skill labs. Having a massed curriculum was associated with a statistically significant increase in exposure to radiation oncology (*p* = 0.009) despite the overall poor exposure.

**Fig. 1:**
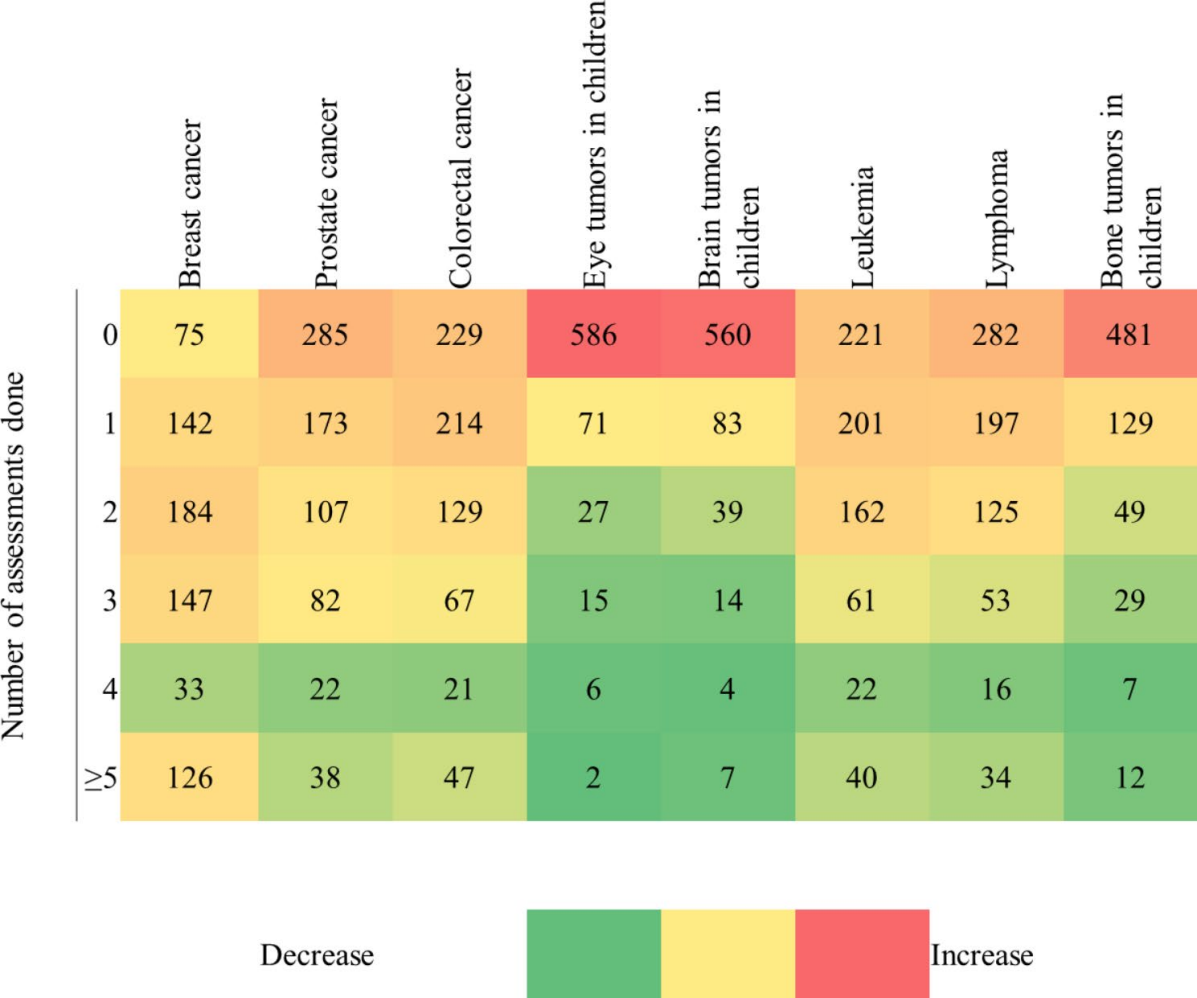
Heatmap showing the frequency of assessment of common malignancies by 2021–2022 graduates: A national study, Sudan, 2022 (n = 707)

Medical and surgical oncology were better, as 317 (44.8%) and 304 (43.0%) of participants reported attending 1–5 h of clinical rounds or skill labs in these disciplines, respectively. The situation was better for lectures/tutorials. However, the overall pattern was maintained. Palliative and radiation oncology received the least attention: 349 (49.4%) and 375 (53.0%) participants reported zero hours of lectures in both disciplines. Pediatric oncology faired in the middle. - with 247 (34.9%) reporting zero hours, and 275 (38.9%) reported 1–5 h. Didactic teaching was best in medical and surgical oncology, as 304 (43.0%) and 318 (45.0%) of participants reported 1–5 h in both disciplines, respectively. Having a massed curriculum was associated with a statistically significant increase in hours dictated to radiation (*p* < 0.001) and palliative oncology (*p* = 0.035) (Table 3). Clinical assessment of common malignancies varied and showed the lack of emphasis on pediatric tumors. Approximately 578 (81.8%) never assessed eye tumors, 554 (78.4%) never assessed brain tumors in children and 475 (67.2%) never assessed pediatric bone tumors (Fig. 1).

**Table 3:** Association between the type of oncology curriculum and hours spent by the graduates in various oncologic disciplines

Characteristic	Oncology curriculum			
	Total, N = 707	Massed, N = 172^1^	Spaced out, N = 535^1^	*p*-value^2^
**Skill labs/clinical rounds in surgical oncology**				0.086
0 hrs	243 (34.4%)	66 (38%)	177 (33%)	
1–5 hrs	304 (43.0%)	61 (35%)	243 (45%)	
6–10 hrs	87 (12.3%)	24 (14%)	63 (12%)	
11–15 hrs	25 (3.5%)	4 (2.3%)	21 (3.9%)	
16–20 hrs	13 (1.8%)	6 (3.5%)	7 (1.3%)	
> 20 hrs	35 (5.0%)	11 (6.4%)	24 (4.5%)	
**Skill labs/clinical rounds in medical oncology**				**0.006**
0 hrs	231 (32.7%)	39 (23%)	192 (36%)	
1–5 hrs	317 (44.8%)	80 (47%)	237 (44%)	
6–10 hrs	93 (13.2%)	32 (19%)	61 (11%)	
11–15 hrs	37 (5.2%)	10 (5.8%)	27 (5%)	
16–20 hrs	14 (2.0%)	4 (2.3%)	10 (1.9%)	
> 20 hrs	15 (2.1%)	7 (4.1%)	8 (1.5%)	
**Skill labs/clinical rounds in pediatric oncology**				> 0.9
0 hrs	360 (50.9%)	85 (49%)	275 (51%)	
1–5 hrs	256 (36.2%)	62 (36%)	194 (36%)	
6–10 hrs	54 (7.6%)	15 (8.7%)	39 (7.3%)	
11–15 hrs	20 (2.8%)	5 (2.9%)	15 (2.8%)	
16–20 hrs	9 (1.3%)	3 (1.7%)	6 (1.1%)	
> 20 hrs	8 (1.1%)	2 (1.2%)	6 (1.1%)	
**Skill labs/clinical rounds in radiation oncology**				**0.009**
0 hrs	509 (72.0%)	109 (63%)	400 (75%)	
1–5 hrs	146 (20.7%)	47 (27%)	99 (19%)	
6–10 hrs	34 (4.8%)	12 (7.0%)	22 (4.1%)	
11–15 hrs	8 (1.1%)	1 (0.6%)	7 (1.3%)	
16–20 hrs	5 (0.7%)	0 (0%)	5 (0.9%)	
> 20 hrs	5 (0.7%)	3 (1.7%)	2 (0.4%)	
**Skill labs/clinical rounds in palliative oncology**				0.7
0 hrs	474 (67.0%)	108 (63%)	366 (68%)	
1–5 hrs	163 (23.1%)	46 (27%)	117 (22%)	
6–10 hrs	49 (6.9%)	13 (7.6%)	36 (6.7%)	
11–15 hrs	14 (2.0%)	4 (2.3%)	10 (1.9%)	
16–20 hrs	2 (0.3%)	0 (0%)	2 (0.4%)	
> 20 hrs	5 (0.7%)	1 (0.6%)	4 (0.7%)	
**Daycare in the chemotherapy unit**				0.094
0 hrs	509 (72.0%)	113 (66%)	396 (74%)	
1–5 hrs	146 (20.7%)	45 (26%)	101 (19%)	
6–10 hrs	30 (4.2%)	9 (5.2%)	21 (3.9%)	
11–15 hrs	11 (1.6%)	2 (1.2%)	9 (1.7%)	
16–20 hrs	5 (0.7%)	0 (0%)	5 (0.9%)	
> 20 hrs	6 (0.8%)	3 (1.7%)	3 (0.6%)	
**Lectures/tutorials in surgical oncology**				0.11
0 hrs	119 (16.8%)	25 (15%)	94 (18%)	
1–5 hrs	318 (45.0%)	68 (40%)	250 (47%)	
6–10 hrs	142 (20.1%)	44 (26%)	98 (18%)	
11–15 hrs	47 (6.6%)	10 (5.8%)	37 (6.9%)	
16–20 hrs	22 (3.1%)	5 (2.9%)	17 (3.2%)	
> 20 hrs	59 (8.3%)	20 (12%)	39 (7.3%)	
**Lectures/tutorials in medical oncology**				**0.005**
0 hrs	112 (15.8%)	15 (8.7%)	97 (18%)	
1–5 hrs	304 (43.0%)	70 (41%)	234 (44%)	
6–10 hrs	167 (23.6%)	42 (24%)	125 (23%)	
11–15 hrs	61 (8.6%)	23 (13%)	38 (7.1%)	
16–20 hrs	16 (2.3%)	5 (2.9%)	11 (2.1%)	
> 20 hrs	47 (6.6%)	17 (9.9%)	30 (5.6%)	
**Lectures/tutorials in pediatric oncology**				0.15
0 hrs	247 (34.9%)	48 (28%)	199 (37%)	
1–5 hrs	275 (38.9%)	68 (40%)	207 (39%)	
6–10 hrs	114 (16.1%)	32 (19%)	82 (15%)	
11–15 hrs	32 (4.5%)	12 (7%)	20 (3.7%)	
16–20 hrs	11 (1.6%)	3 (1.7%)	8 (1.5%)	
> 20 hrs	28 (4.0%)	9 (5.2%)	19 (3.6%)	
**Lectures/tutorials in radiation oncology**				**< 0.001**
0 hrs	375 (53.0%)	66 (38%)	309 (58%)	
1–5 hrs	212 (30.0%)	64 (37%)	148 (28%)	
6–10 hrs	67 (9.5%)	25 (15%)	42 (7.9%)	
11–15 hrs	22 (3.1%)	5 (2.9%)	17 (3.2%)	
16–20 hrs	15 (2.1%)	6 (3.5%)	9 (1.7%)	
> 20 hrs	16 (2.3%)	6 (3.5%)	10 (1.9%)	
**Lectures/tutorials in palliative care**				**0.035**
0 hrs	349 (49.4%)	68 (40%)	281 (53%)	
1–5 hrs	226 (32.0%)	63 (37%)	163 (30%)	
6–10 hrs	77 (10.9%)	23 (13%)	54 (10%)	
11–15 hrs	28 (4.0%)	8 (4.7%)	20 (3.7%)	
16–20 hrs	12 (1.7%)	6 (3.5%)	6 (1.1%)	
> 20 hrs	15 (2.1%)	4 (2.3%)	11 (2.1%)	

^1^n (%)

^2^Pearson’s Chi-squared test

### Graduates’ self-reported confidence in various oncologic competencies and its predictors

**Fig. 2:**
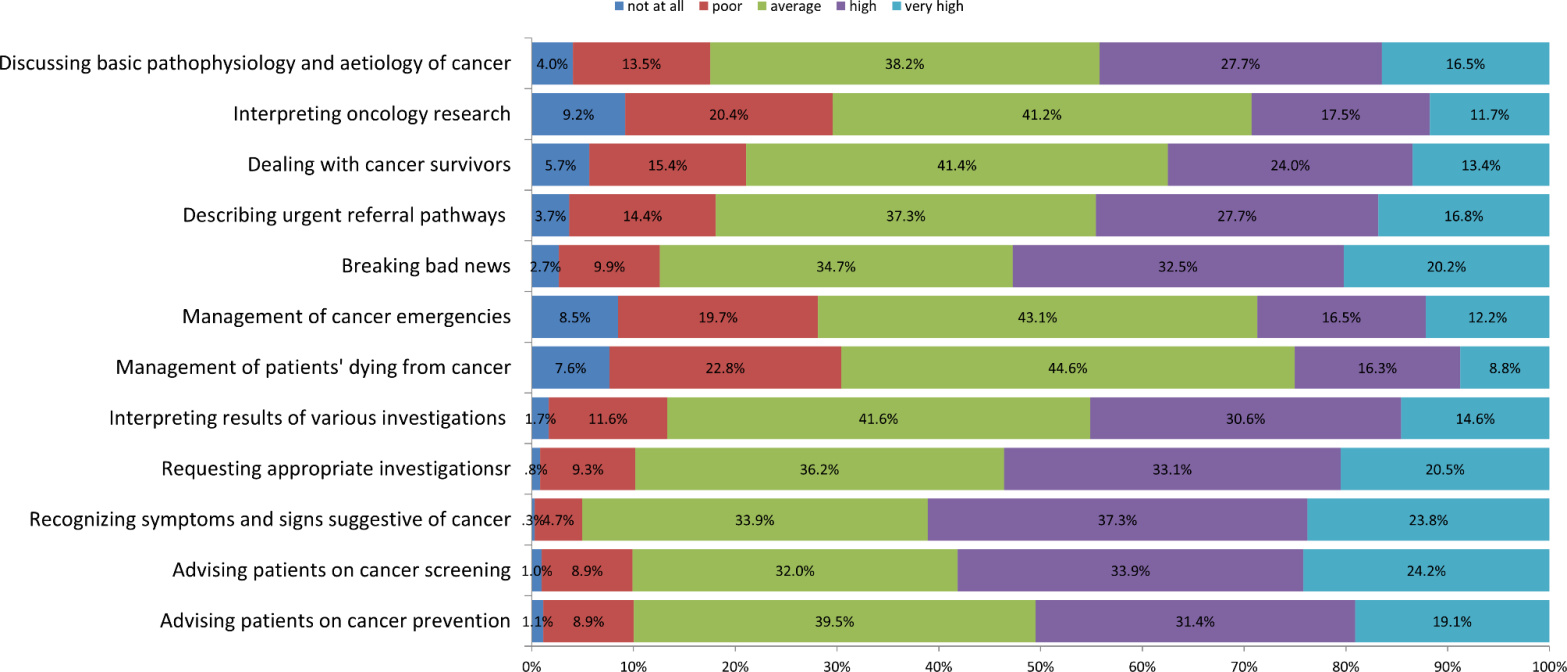
Stacked bar showing graduates’ self-reported confidence in various oncologic competencies – A national study, Sudan, 2022 (n = 707)

Participants were asked to rate their confidence in various oncologic competencies. The majority perceived average confidence or higher in all competencies. More than 25% of graduates reported a lack of confidence in the following competencies: interpreting oncology research, 209 (29.6%); managing cancer emergencies, 199 (28.2%); managing dying patients, 215 (30.4%); and dealing with cancer survivors, 39 (5.6%) (Fig. 2).

Confidence in oncology-related skills was higher among graduates who observed multidisciplinary care, witnessed shared decision-making, and were assessed in oncology-related knowledge, history-taking from cancer patients, cancer examination, and counselingcancer patients. Other associated factors were being male and the number of cancer patients assessed during undergraduate years (*p* value < 0.05). Interestingly, the presence of an oncology course in the medical school did not significantly affect confidence (*p* value = 0.7). Table 4.

**Table 4:** Predictors of self-reported oncology competence among graduates of governmental medical schools

Characteristic	N = 707^1^	*p*-value^2^
**Observing multidisciplinary care**		0.002
No	40 ± 9	
Yes	42 ± 8	
**Observing the shared decision-making process between doctors and cancer patients**		**< 0.001**
No	39 ± 9	
Yes	43 ± 8	
**Assessment of history taking from cancer patient**		**< 0.001**
No	38 ± 9	
Yes	41 ± 9	
**Assessment of examining a cancer patient**		**< 0.001**
No	38 ± 9	
Yes	41 ± 9	
**Assessment of breaking bad news to cancer patients**		**< 0.001**
No	39 ± 9	
Yes	42 ± 8	
**Assessment of counseling cancer patients**		**< 0.001**
No	39 ± 9	
Yes	42 ± 8	
**Assessment of oncology knowledge**		**0.002**
No	39 ± 9	
Yes	41 ± 8	
**Sex**		**0.039**
Female	40 ± 9	
Male	41 ± 9	
**Total number of assessed cancer cases**		**0.021**
10 times or less	40 ± 9	
11–20 times	42 ± 8	
21–30 times	41 ± 9	
31 times and more	47 ± 8	
**Having oncology courses**		0.7
Courses	41 ± 9	
No courses	41 ± 9	

^1^Confidence score: Mean ± SD

^2^Welch Two Sample t-test; One-way ANOVA

## Discussion

Surveying graduates of 17 public medical schools in Sudan revealed poor exposure to oncology at undergraduate level. Only four medical schools (University of Bahri, University of Shendi, Omdurman Islamic University of males and that of females) use a massed curriculum approach to teach oncology and have a detailed undergraduate oncology course. Other universities use a spaced-out curriculum approach, and oncology is taught as part of other medical specialties without clear objectives and hours dictated to oncology.

32% of the surveyed participants showed interest in oncology careers. This is promising given the shortage of cancer care providers [20]. Sudan has a clinical oncology training program under the Sudan Medical Specialization Board (SMSB) but no surgical or medical oncology training programs. Graduates interested in oncology can either specialize in clinical oncology or apply for training outside Sudan. It was observed that graduates of universities with a massed oncology curriculum were less likely to be interested in pursuing a career in oncology, and this difference was statistically significant. A large national retrospective study in the UK found no association between length of specialty exposure at the undergraduate level and pursuing that specialty [21]. It is worth emphasizing that specialty choices and interests are multifactorial. However, the negative association between oncology course and interest should be investigated to determine the course-related factors that discourage interest in oncology and solve this issue.

In spite of the general popularity of massed education and its claimed superiority [22], Sudanese graduates preferred the spaced out approach of teaching oncology. Students’ preferences should be taken into consideration when developing an oncology curriculum. However, the efficacy of each approach in Sudan should also be considered after objective assessment.

The majority of students attended 1–5 h of oncology-related sessions in surgery and medicine. However, most of them never got exposed to pediatric, radiation and palliative oncology as well as daycare chemotherapy units. This was reflected in their clinical experience of oncology. Common pediatric tumors, such as brain, eye and bone tumors, were never assessed by almost two-thirds of graduates. Even common tumors such as prostate cancer, colorectal cancer, leukemia and lymphoma were not assessed by more than one-third of the participants. This is consistent with the situation in other lower middle-income countries (LMICs) [9, 14] and may be translated into missing cancer diagnoses and false directions of patients by general practitioners and primary healthcare workers [23]. The difference in exposure to radiation and palliative oncology compared with medical and surgical oncology was also noted in Australia, where students reporting never attending palliative or radiation oncology clinics was double that of students never attending medical or surgical oncology clinics [24]. This was expected, as palliative and radiation oncology are considered very specialized for medical students [25]. Having a massed curriculum was associated with increased exposure to radiation and palliative oncology. It is argued that the basics of radiation oncology are essential for general practice [25] and important in appreciating multidisciplinary care [5]. The relevance of radiation as well as palliative oncology for undergraduate students in Sudan should be discussed by Sudanese specialists and medical education experts.

Graduates had poor exposure to multidisciplinary care and shared decision making in oncology, and there was no statistically significant difference in exposure between universities with a massed curriculum and those with a spaced-out curriculum. Studies from other parts of the globe have highlighted the need to focus on the ethical aspects of cancer care [26]. Sudan lacks multidisciplinary cancer care units and guidelines that organize multidisciplinary care [15]. Assessment was least likely to target breaking bad news or counseling cancer patients. Assessment has a major impact on students’ learning; if students are not assessed in terms of communication skills, they are less likely to develop them [27]. This is concerning, as effective communication skills reduce the psychological burden associated with cancer diagnosis [28]. Graduates should be aware of the amount of news they break before referring the patients for oncologists.

In spite of the general poor exposure, graduates were confident in their skills in various oncologic competencies. This was different from the United States, where students reported good exposure but lack of confidence in oncology care [6]. Overestimation and underestimation are related to the perception of competence [29], which is affected by exposure. The curriculum approach was not associated withself-reported confidence.

It is important to remember that confidence in competence does not reflect the actual competence. Indeed, students tend to overestimate their clinical skills [30]. Taking this into consideration, reporting a lack of confidence in the management of cancer emergencies is quite concerning, especially in Sudan, where cancer emergencies are managed in general hospitals, not in oncology hospitals. Cancer is the fifth leading cause of death in Sudan general hospitals [3]. Therefore, a lack of confidence in the management of dying patients is alarming. Graduates were not confident in their skills in interpreting oncology research, which is consistent with a study that assessed knowledge, attitude and practice toward evidence-based medicine among Sudanese medical students [31].

This is the first study to describe undergraduate oncology curriculum in Sudan and one of the few studies described on undergraduate oncology curriculum in Africa [9, 14]. This was a nationwide large-scale study that covered 17 public medical schools using a stratified random sampling approach, thus limiting response bias. We compared universities with massed and spaced-out oncology course. Surveying fresh graduates is a a strength of this study because they are more likely to reflect undergraduate oncology exposure without missing details compared to surveying final-year students.

The main limitation of this study is the use of self-reported confidence to measurecompetence. This approach is common in medical education [9]. Nevertheless, we have emphasized the subjectivity of self-reported confidence in competence and did not associate it with actual competence throughout our discussion. We also focused on discussing other parts of the questionnaire that reflect the graduate’s exposure and opinion on oncology. Objective description and assessment of the oncology curriculum was limited by its absence or lack of clarity in most universities.

## Conclusion

This study reflected poor exposure to oncology at the undergraduate level in Sudan public medical schools. This poor exposure was coupled with high subjective confidence in oncology competencies. Graduates showed preference of spaced-out curriculum. Massed oncology curriculum was associated with formal assessment in oncology related competencies and better exposure to some disciplines like radiation and palliative oncology. Nonetheless, it was associated with decreased interest in oncology career. Graduates were generally confident in their oncology related competencies. The majority of graduates reported the need of a national oncology curriculum. We recommend future research objectively assess graduates’ competence in oncology, and compare massed and spaced-out approaches. The vague nature of oncology curriculum in the majority of universities is concerning. Sudanese specialists and educators are encouraged to use delphi approach to determine essential oncologic competencies that fit the local context.

### Electronic supplementary material

Below is the link to the electronic supplementary material.

**Additional file 1: Supplementary table 1**. Details of the sampling

## Data Availability

All data supporting the findings of this article will immediately be available upon request from the corresponding author.

## Abbreviations

ESO: European School of Oncology
GLOBOCAN: Global Cancer Incidence, Mortality and Prevalence
HIC: High-income countries
LMIC: Lower- and middle-income countries
