# Supplementary material for: Undergraduate oncology education in Sudanese public medical schools; a national cross-sectional study
Source: BMC Med Educ. 2023 Dec 10;23:940. doi: 10.1186/s12909-023-04883-0 (PMC10712034; doi:10.1186/s12909-023-04883-0)
Supplement: Supplementary file 1 — Additional file 1: Supplementary table 1. Details of the sampling [file 12909_2023_4883_MOESM1_ESM.docx]

**Undergraduate oncology education in Sudanese public medical schools; a national cross-sectional study**

**Supplementary table 1. Details of the sampling**

| University | State | Availability of oncology center in the State | Total number of included graduates | Calculated sample size | Received responses | Response rate |
| --- | --- | --- | --- | --- | --- | --- |
| University of Albutana | Gezira | Yes | 125 | 36 | 30 | 83.33% |
| Bakht Alruda University | White Nile | No | 124 | 36 | 30 | 83.33% |
| Sinnar University | Sinnar | No | 125 | 36 | 29 | 80.56% |
| Shendi University | River Nile | Yes | 121 | 35 | 35 | 100% |
| University of Gadarif | Gadarif | Yes | 140 | 41 | 35 | 85.37% |
| Alfashir University | North Darfur | Yes | 150 | 44 | 36 | 81.82% |
| University of Elimam Elmahdi | White Nile | No | 167 | 49 | 40 | 81.63% |
| University of Kassala | Kassala | No | 222 | 65 | 53 | 81.54% |
| Red Sea University | Red Sea | Yes | 158 | 46 | 37 | 80.43% |
| University of Kordofan | North Kordofan | Yes | 114 | 33 | 28 | 84.84% |
| Alzaiem Alazhari University | Khartoum | Yes | 187 | 54 | 44 | 81.48% |
| University of Bahri | Khartoum | Yes | 266 | 77 | 63 | 81.81% |
| Neelain University | Khartoum | Yes | 225 | 65 | 52 | 80.00% |
| Omdurman Islamic University of males | Khartoum | Yes | 126 | 37 | 31 | 83.78% |
| Omdurman Islamic University of females | Khartoum | Yes | 172 | 50 | 43 | 86.00% |
| Gezira University | Gezira | Yes | 207 | 60 | 48 | 80.00% |
| University of Khartoum | Khartoum | Yes | 283 | 82 | 73 | 89.02% |
